# Plasma metabolomic profiling in two rabbit lines divergently selected for intramuscular fat content

**DOI:** 10.1038/s42003-023-05266-3

**Published:** 2023-08-31

**Authors:** Agostina Zubiri-Gaitán, Agustín Blasco, Pilar Hernández

**Affiliations:** Institute for Animal Science and Technology, Universitat Politècnica de València, Valencia, Spain

**Keywords:** Quantitative trait, Animal breeding, Metabolomics

## Abstract

This study provides a thorough comparison of the plasma metabolome of two rabbit lines divergently selected for intramuscular fat content (IMF). The divergent selection led to a correlated response in the overall adiposity, turning these lines into a valuable animal material to study also the genetics of obesity. Over 900 metabolites were detected, and the adjustment of multivariate models, both discriminant and linear, allowed to identify 322 with differential abundances between lines, which also adjusted linearly to the IMF content. The most affected pathways were those of lipids and amino acids, with differences between lines ranging from 0.23 to 6.04 standard deviations, revealing a limited capacity of the low-IMF line to obtain energy from lipids, and a greater branched-chain amino acids catabolism in the high-IMF line related to its increased IMF content. Additionally, changes in metabolites derived from microbial activity supported its relevant role in the lipid deposition. Future research will focus on the analysis of the metabolomic profile of the cecum content, and on the integration of the several -omics datasets available for these lines, to help disentangle the host and microbiome biological mechanisms involved in the IMF deposition.

## Abstract (web summary)

Comprehensive metabolomic profiling of plasma samples from two rabbit lines that were divergently selected for intramuscular fat content highlights the contribution of host genetics and microbial-derived metabolites to obesity or lipid deposition.

## Introduction

The intramuscular fat content (IMF) is a key parameter of meat quality^1^, influencing multiple characteristics like tenderness, juiciness, water-holding capacity, etc.^2^. At the Universitat Politècnica de València, a divergent selection experiment for IMF in *Longissimus Thoracis et Lumborum* (LTL) muscle was performed in rabbits^3,4^. As a result, two rabbit lines were created, one with high-IMF content (H) and the other with low-IMF content (L). The selection started from the same base population, and both lines were contemporarily reared under the same environmental conditions and were fed the same diet, meaning that the differences found between them can be directly attributed to their genetic composition. This selection led to a correlated response in the overall adiposity, making these lines a valuable animal material to study also the genetics of obesity.

In the last few years, several -omics techniques have been used to obtain a better characterization of meat quality^5^, focusing on several of the aforementioned characteristics, including IMF^6–10^. Among those techniques, metabolomic has gained special attention and is considered a key factor in next-generation phenotyping approaches^11,12^. Metabolites are the set of organic molecules of small molecular mass found in a biological sample. They are the last response of biological systems to genetic and environmental effects before the expression of the phenotype, and are therefore considered as an intermediate phenotype^13^. The analysis of the metabolomic profile has given some valuable insights regarding the biological mechanisms controlling different meat and carcass quality traits^14–17^. For instance, urinary content of bile acids and steroids was associated to marbling in cattle, although that association varied depending on the average daily gain of the animal, the sampling time, and the diet^17^. Li et al.^18^ on the other hand, found several plasma metabolites related to carcass merit traits in beef cattle, with each one accounting only for 0.8–2.71% of the phenotypic variance, reflecting the complex nature of the traits. In pigs, higher plasma concentration of branched-chain amino acids^19^ and serum concentration of L-carnitine^15^ were associated to greater IMF content, and were even proposed as biomarkers. The former studies evidenced the variety of metabolic pathways affecting the IMF content and other meat and carcass quality traits. A metabolomic analysis on these divergent lines will allow us to take a closer look into the genetically determined mechanisms responsible for fat deposition, giving relevant insights that could be extrapolated to the study of obesity.

Genomic and metagenomic studies have already been performed on these lines evidencing, on one hand, several genes related to the IMF content and its fatty acid composition^20,21^ and, on the other hand, the importance of the microbiome activity in the lipid deposition in muscle and other adipose tissues^22^. In this study, an untargeted metabolomic approach was used to identify the genetically induced changes in the plasma metabolomic profile of the IMF divergent lines, disclosing the metabolic routes involved in their differentiation. The results obtained revealed a large and varied response to selection, affecting mainly the metabolisms of lipids and amino acids, and to a lesser extent those of carbohydrates and vitamins A and E. A general increase of lipids was observed in the plasma of the L line, which suggested an impaired β-oxidation of fatty acids. These results indicate a limited capacity of the L line to obtain energy from lipids, consequently reducing their uptake and re-esterification in muscle and adipose tissue. The most relevant results among the amino acids metabolism were related to the BCAA, which suggested a lower degradation in the gut of the H line, followed by a greater catabolism by the host. Additionally, these results supported the importance of microbiome activity, relating several metabolites derived from the gut microbiome metabolism to the lipid deposition and, more importantly, validating some of the results found in the metagenomic study previously performed^22^.

The effect of the metabolites on the development of the trait was inferred based on functions extracted from the literature, and from previous results found on these divergent lines. No functional validation was performed, which can represent a limitation of the present study. Future research will be focused on the analysis of the metabolomic profile of the cecum content, and on the integration of the several -omics datasets available for these lines, which will help disentangle the host and microbiome biological mechanisms and their interplay involved in the IMF deposition.

## Results and discussion

Metabolomics has proven to be a powerful tool, and the analysis performed on these lines yielded many metabolites related to the intramuscular fat deposition, which allowed us to make important inferences over the biological mechanisms that led to a greater or lower fat deposition. In this study, after the data processing, 920 metabolites were kept for further analysis (242 positive-early, 168 positive-late, 399 negative, and 111 polar), from which 789 were known entities. The reference metabolites chosen for the *alr* transformation of each dataset, following the methodology described by Greenacre et al.^23^, were: proline for the positive-early dataset, sphingomyelin (d18:2/24:1, d18:1/24:2) for the positive-late dataset, *N*-acetyl valine for the negative dataset, and glucose for the polar dataset.

**Fig. 1:**
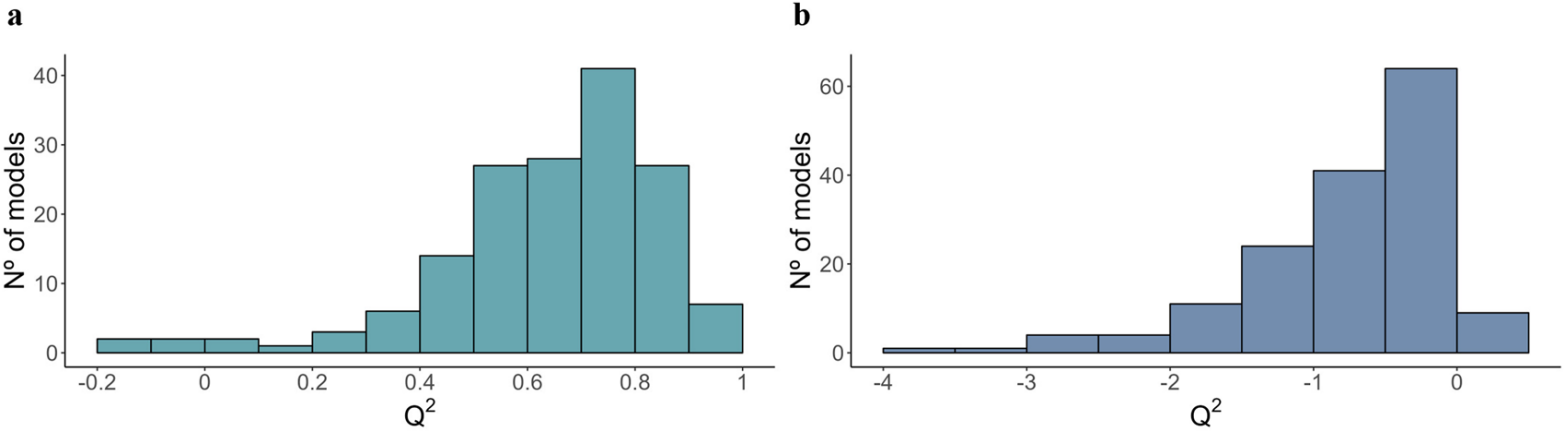
Adjustment parameters of the PLS models. Distribution of all the Q^2^ values obtained from the PLS models adjusted during the cross-validation procedure with (**a**) true data, and with (**b**) permuted data.

The adjustment parameters of the PLS-DA models adjusted with true and permuted data are shown in Table 1 (supplementary data 1 and 2), while those of the PLS models are shown in Fig. 1a (true data; supplementary data 3) and Fig. 1b (permuted data; supplementary data 4). As it can be seen in the average misclassification table, the PLS-DA models showed a good classification ability of around 95%, while that of the permutation test was around 50%, evidencing the robustness of the PLS-DA models adjusted with the original data (Table 1). Similarly, the Q^2^ parameter estimated for the PLS models showed a good prediction accuracy (mean: 65%; Fig. 1a), and this was supported by the prediction accuracy obtained from the permutation test (Fig. 1b). These results showed that there was an important correlated response to selection on the plasma metabolome of the lines.

**Table 1:** Adjustment parameters obtained from the predictions of the PLS-DA model during the CMV procedure.

Misclassification table with true data			Misclassification table with permuted data		
	^1^H (%)	^1^L (%)		^1^H (%)	^1^L (%)
^1^H	96.7	3.3	^1^H	54.8	45.2
^1^L	5.4	94.6	^1^L	49.2	50.8

^*1*^*H* high-IMF line, *L* low-IMF line.

**Fig. 2:**
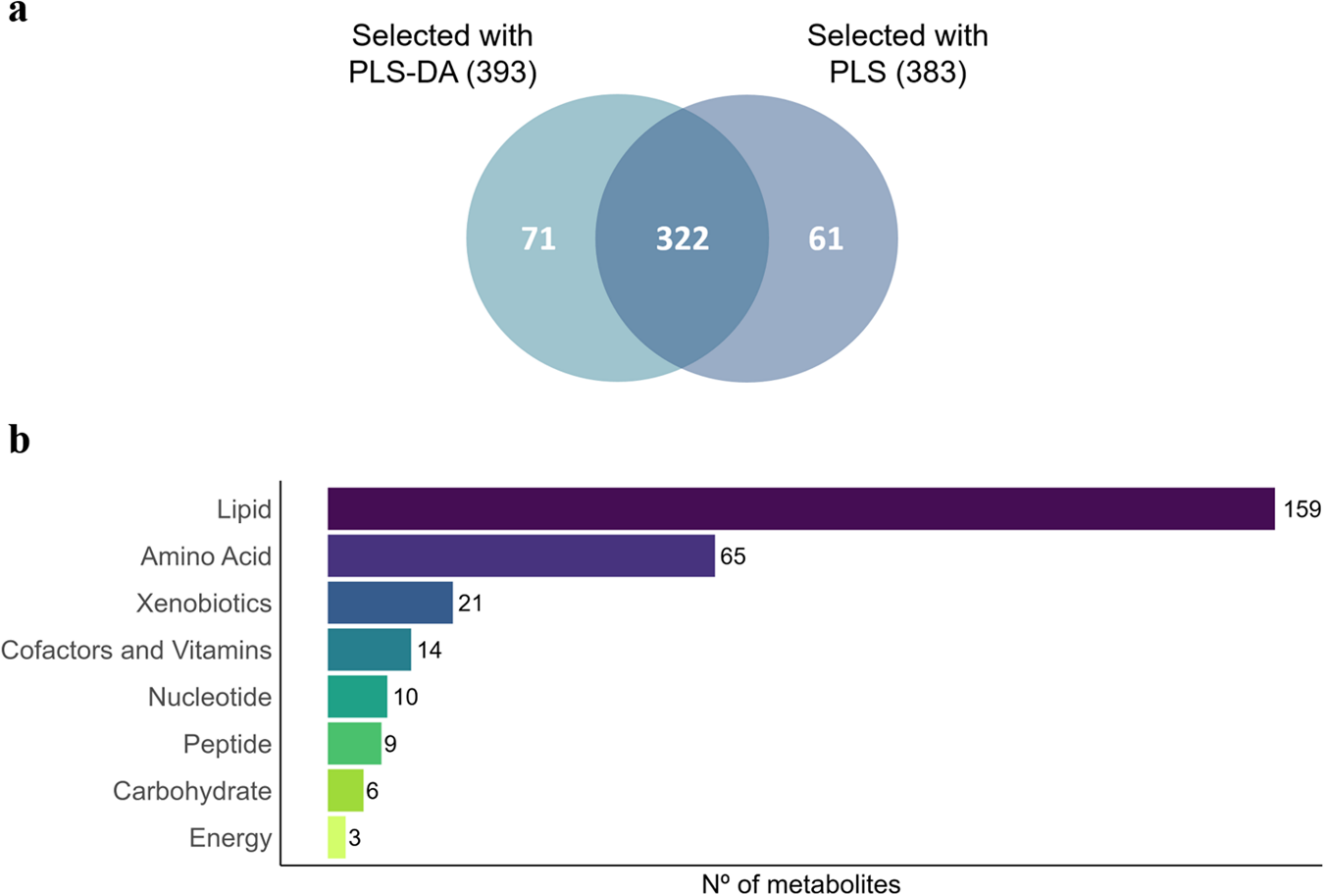
Summary of the relevant metabolites obtained with the PLS and PLS-DA models. **a** Venn diagram of the metabolites selected using PLS-DA and PLS approaches; **b** classification of the 322 metabolites selected.

Once the PLS-DA and PLS models were developed and their performance was validated, the relevant metabolites were selected according to the procedure previously described. However, 16 out of the 160 PLS models that presented a bad prediction accuracy (*Q*^2^ < 0.4) were not considered for the selection (Fig. 1a). Supplementary data 5 shows the complete metabolomic profile obtained, together with a summary of the results obtained from the PLS-DA and PLS models. In a divergent selection experiment, the lines are created from the same base population, are fed the same diet, and are contemporarily reared under the same environmental conditions, which means that the differences found between them are expected to be mainly due to their genetic composition. However, those differences can also be due to genetic drift occurred during the selection procedure. On the other hand, even though the PLS analysis associates metabolites to the IMF trait (independently of the line), extreme values of this trait are likely due to environmental causes. Therefore, considering the metabolites that were selected in both PLS-DA and PLS approaches corroborates that those differences are most likely due to genetic causes. Finally, 322 metabolites that presented differences between the lines (selected with the PLS-DA) and adjusted linearly to the IMF trait (selected with the PLS) were considered as a correlated response to selection (Fig. 2a).

The 322 selected metabolites are shown in supplementary data 6, together with the metabolic pathways in which they are involved. Considering the number of metabolites found, the most affected metabolisms were those of lipids (159) and amino acids (65). The remaining metabolites were involved in the metabolism of xenobiotics (21), cofactors and vitamins (14), nucleotides (10), peptides (9), carbohydrates (6), and energy (3) (Fig. 2b and supplementary data 6). Finally, there were 33 metabolites that could not be identified and 2 that were partially characterized molecules. This large and varied response to selection found in the plasma metabolome agrees with the large polygenic component found in a genome-wide association study (GWAS) previously performed on these lines^20^.

### Lipid metabolism

**Fig. 3:**
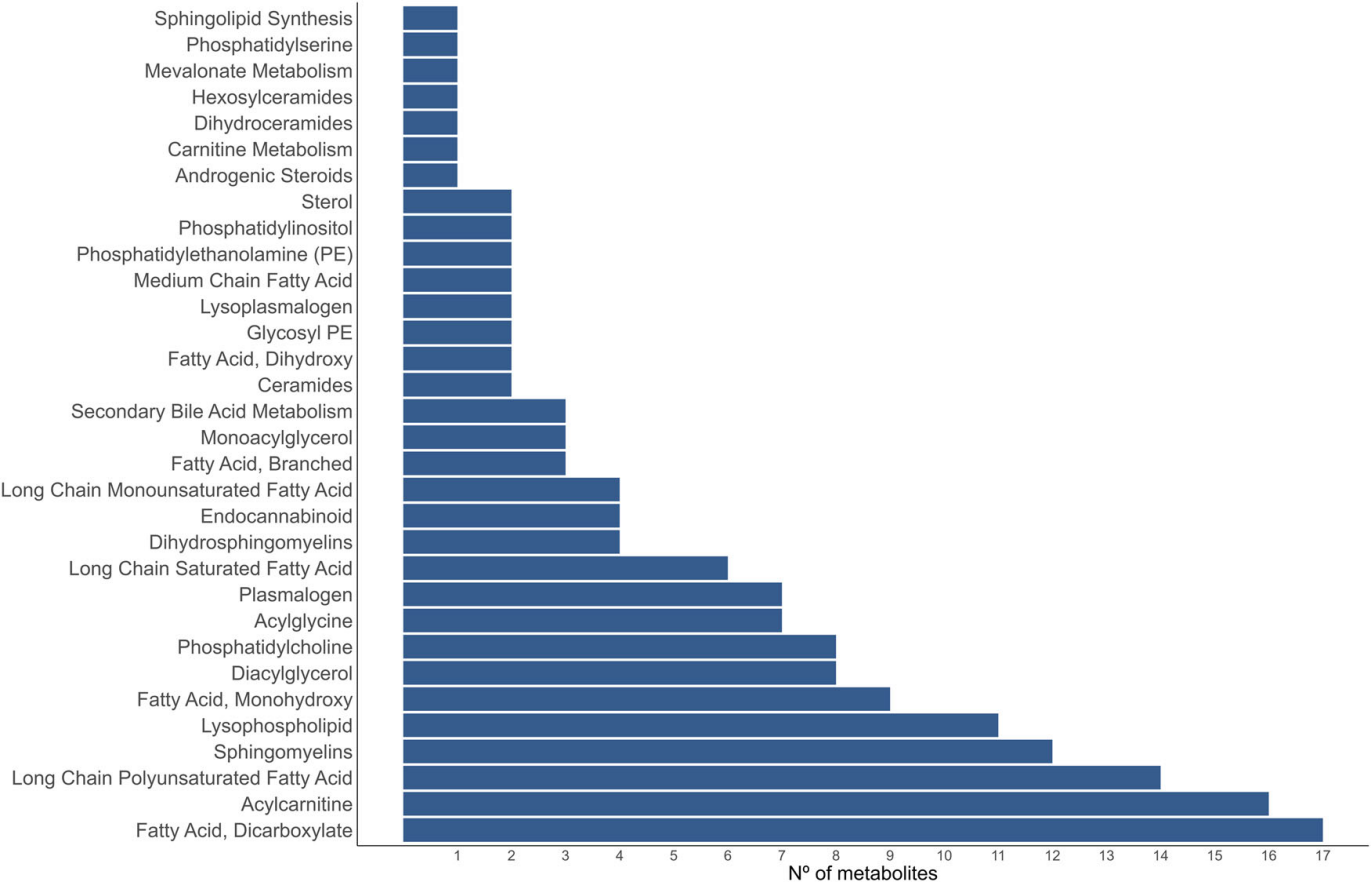
Lipid sub-pathways. Number of differentially abundant metabolites found for each lipid sub-pathway.

The lipid metabolites found can be categorized as shown in Fig. 3 (supplementary data 6). Most of these metabolites were more abundant in the plasma of the L line (127 out of 159; see supplementary data 6) and the most relevant were non-esterified fatty acids (NEFAs), including medium-chain, branched-chain, long-chain (LC) saturated, LC monounsaturated, and LC polyunsaturated fatty acids (0.51 to 1.27 SD, *P*_0_ ≥ 0.96), monoacylglycerols (MAGs; 0.73 to 0.94 SD, *P*_0_ ≥ 0.99), diacylglycerols (DAGs; 0.84 to 6.04 SD, *P*_0_ = 1), acylcarnitines (0.29 to 1.56 SD, *P*_0_ ≥ 0.84), acylglycines (0.41 to 1.50 SD, *P*_0_ ≥ 0.92), and dicarboxylic acids (0.27 to 1.44 SD, *P*_0_ ≥ 0.83). Other changes in the lipid metabolism were reflected in the greater abundance in the L line of cholesterol (1.11 SD, *P*_0_ = 1), cholesterol sulfate (0.89 SD, *P*_0_ = 1), secondary bile acids (0.61 to 1.01 SD, *P*_0_ ≥ 0.98), sphingomyelins (0.73 to 1.62 SD, *P*_0_ ≥ 0.91) and plasmalogens (0.44 to 1.28 SD, *P*_0_ ≥ 0.94), and the lower abundance of lysophospholipids (0.74 to 1.53 SD, *P*_0_ ≥ 0.99). The differences in cholesterol and bile acids was also observed in the plasma of the L line on the 10th generation of selection^24^. Additionally, previous experiments also found greater amount of triglycerides in the plasma of the L line^24,25^.

Bile acids are polar derivatives of cholesterol and play an essential role in lipid digestion and absorption in the intestine^26^. The greater amount of cholesterol and triglycerides previously observed can be related to the greater amount of bile acids, although further analyses are needed to confirm this theory. Despite this greater amount of cholesterol and triglycerides, the differences found in all the lipid metabolites mentioned above, may suggest a limited capacity of the L line to obtain energy from those lipids, causing their accumulation together with that of their catabolic products, as will be discussed later. The NEFAs and MAGs found in this experiment are products of the activity of the lipoprotein lipase (LPL) on the triglycerides^27^. Greater circulating NEFAs were also found in pigs with lower IMF content caused by the CC and CT genotypes of the LEPR gene; however, the difference was awarded to a greater fat mobilization in those pigs^28^. Other products of the hydrolysis of triglycerides, like the glycerol and glycerol-3-phosphate, were also relevant in the discrimination, with 96% (glycerol) and 98% (glycerol-3-phosphate) probability of being more abundant in the L line (supplementary data 6). Even though those metabolites did not overcome the 80% threshold in both models (supplementary data 5), the statistical evidence, together with their close relationship to NEFAs and MAGs, suggest that they should be also considered in the discussion.

The fatty acids obtained from the triglycerides’ hydrolysis would then be taken up by the cells, where they would be oxidized for energy, through β-oxidation, or reesterified for storage^29^. The β-oxidation of fatty acids takes place inside the mitochondria, where long-chain fatty acids cannot be passively transported through the plasma membrane and must be attached to carnitine, forming acylcarnitines, to be transported through the carnitine shuttle^29^. In this experiment 14 out of the 16 acylcarnitines found were more abundant in the L line. The only exceptions were the short acylcarnitines butyrylcarnitine (0.82 SD, *P*_0_ = 1) and propionylcarnitine (0.74 SD, *P*_0_ = 0.99), which are also related to the branched-chain amino acids (BCAA) metabolism and will be discussed later. Additionally, the carnitine was more abundant in the plasma of the H line (0.74 SD, *P*_0_ = 0.99), which has already been proposed as a biomarker for higher meat quality and IMF content in pigs^15^. The acylcarnitines can increase in the plasma to avoid the accumulation of toxic acyl-COAs in the mitochondria caused by disorders in the β-oxidation. This detoxification mechanism, which has been observed in individuals with type 2 diabetes and insulin resistance, consists in the cross back of the acylcarnitines to the bloodstream, which cause an increased level of plasma acylcarnitines and a decreased level of free carnitine^30^, as observed in these lines. The acylglycines, which are minor metabolites of fatty acids, are also formed to prevent the accumulation of toxic acyl-CoA and, in humans, this detoxification mechanism has been observed in individuals with disorders of short- and medium-chain fatty acid oxidation^31^. Other results supporting this theory are the greater abundance of dicarboxylic acids and DAGs. The dicarboxylic acids are formed from the ω-oxidation of monocarboxylic acids when the β-oxidation of NEFA is impaired^32^, while the increased synthesis of DAGs has been observed when the uptake and oxidation of fatty acids is impaired^33^. The lower β-oxidation in the L line can also be supported by the lower activity of the β-hydroxyacyl-CoA dehydrogenase, an enzyme of the β-oxidation pathway, found in the LTL muscle of the L line in a previous experiment^34^.

The accumulation of triglycerides and their catabolic by-products, together with the accumulation of acylcarnitines, acylglycines and dicarboxylic acids, and the lower carnitine in the plasma L line suggest a reduced uptake in their muscle and other adipose tissues, followed by a lower, and possibly impaired, β-oxidation of fatty acids, which could partially explain its lower fat content.

### Amino acids metabolism

**Fig. 4:**
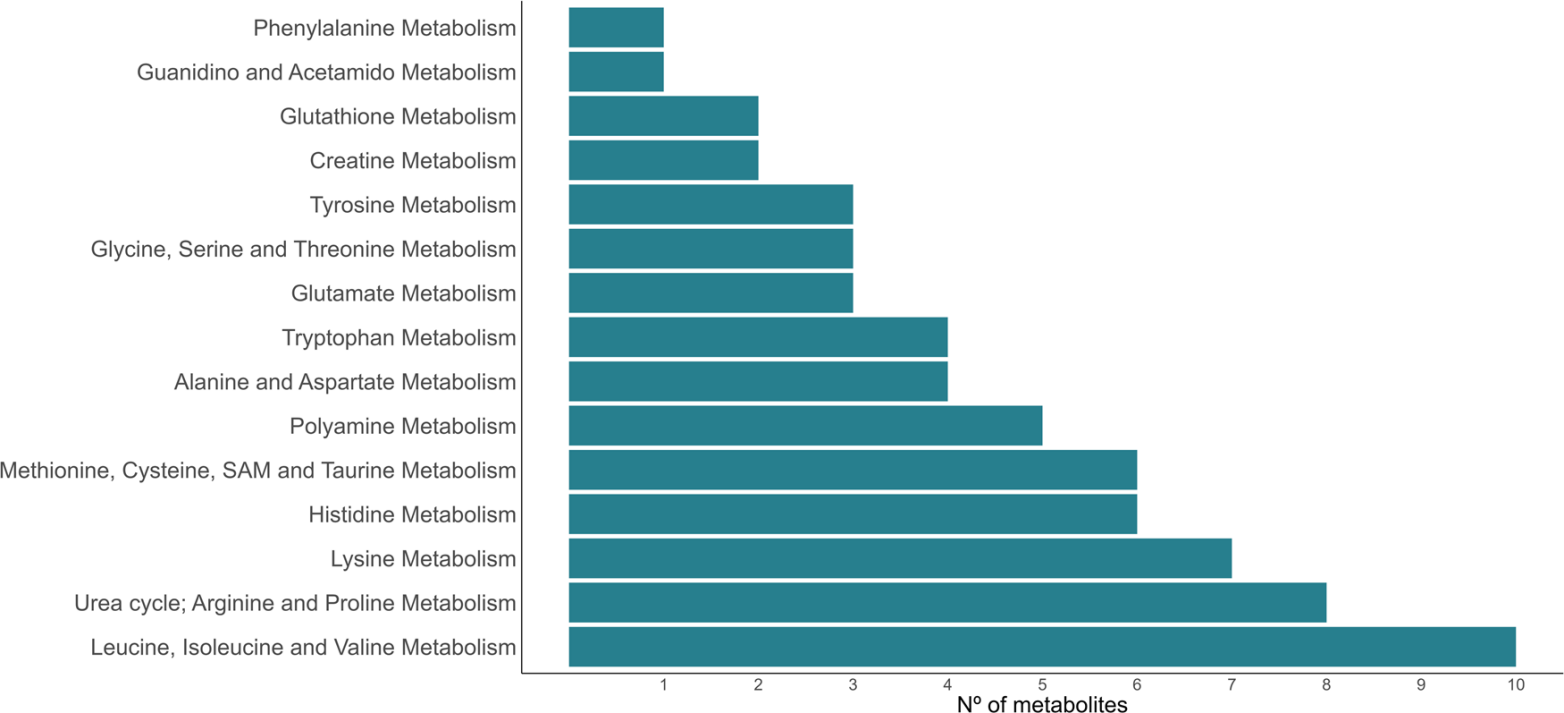
Amino acid sub-pathways. Number of differentially abundant metabolites found for each amino acid sub-pathway.

The metabolites from the amino acids’ metabolism found were involved in several pathways, which can be seen in Fig. 4 (supplementary data 6). Among those pathways, the branched-chain amino acids (BCAA) metabolism stands out (leucine, isoleucine, and valine). The BCAA are essential amino acids that play numerous metabolic and regulatory roles, and have been associated with increased lipolysis, but also with increased lipogenesis^35^. Its blood concentration has been positively associated with IMF content in pigs^19^ and cattle^16^, and with obesity and insulin resistance in humans^36^. In this experiment no differences were found in valine, leucine, and isoleucine, but greater abundances of the modified amino acids (*N*-acetyl leucine, *N*-lactoyl leucine, *N*-lactoyl isoleucine, and *N*-lactoyl valine) were found in the plasma of the H line (0.54–0.75 SD, *P*_0_ ≥ 0.96), together with several metabolites from their catabolism, including the BCAA-associated species butyrylcarnitine and propionylcarnitine mentioned earlier (0.35–1.97 SD, *P*_0_ ≥ 0.88). Exceptionally, the isovalerylglycine and 3-methylcrotonylglycine were more abundant in the plasma of the L line; however, these metabolites are acylglycines, also formed as minor metabolites of the fatty acids, as mentioned earlier. In the GWAS performed on these lines, the BCAT1 gene, that codifies for the first enzyme in the BCAA catabolic pathway expressed in the cytosol, was located in one of the regions associated with the IMF content^20^. This gene has also been associated to obesity in humans in an epigenome-wide association study^37^, where it was found in an hypomethylated state. Additionally, a study in mice showed that the disruption of the BCAT2 gene, expressed in the mitochondria, led to lower BCAA catabolism, which consequently led to a lower fat content^38^. The results found in this experiment suggest a greater BCAA catabolism in the H line that could partially explain its greater IMF content, as observed in humans^39^, although the mechanisms are not entirely understood^36^.

Other changes in the plasma amino acids concentrations included greater abundances in the L line of glycine (1.55 SD, *P*_0_ = 1) and serine (1.04 SD, *P*_0_ = 1), together with metabolites from the creatine metabolism (0.75–1.18 SD, *P*_0_ = 1), lysine metabolism (0.75–1.14 SD, *P*_0_ = 1), the glutamate metabolism (0.37–1.37 SD, *P*_0_ ≥ 0.9), the polyamine metabolism (0.23–1.77 SD, *P*_0_ ≥ 0.8), and the urea cycle, arginine and proline metabolism (0.66–1.38 SD, *P*_0_ ≥ 0.98). On the other hand, the H line had greater plasma abundances of alanine (1.03 SD, *P*_0_ = 1), aspartate (0.81 SD, *P*_0_ = 1), asparagine (0.52 SD, *P*_0_ = 0.96), together with metabolites from the methionine, cysteine, S-adenosyl methionine (SAM) and taurine metabolism (0.54–0.75 SD, *P*_0_ ≥ 0.96). Among those, the arginine metabolism stands out because of the close relationship of arginine and adiposity. Arginine supplementation has been negatively associated with adiposity in obese humans with type-2 diabetes^40^, rats^41, 42^, pigs^43^, and broiler chickens^44^, and has also been shown to retard the progression of atherosclerosis in rabbits fed a high-cholesterol diet^45^. Potential mechanisms has been proposed^46^, which included the enhanced synthesis of cell-signaling molecules like the polyamines, which were also more abundant in the plasma of the L line. Additionally, the study in pigs previously mentioned showed that the arginine supplementation had an effect on the intestinal microbial metabolism^43^. Even though these lines are fed the same diet, the metagenomic analysis performed on these lines found a greater abundance of the *arcA* gene^22^, which codes for a positive regulator of the arginine catabolic pathway^47^. These results could indicate that, despite the same arginine intake, the microbiome composition of the L line could lead to its increased utilization.

### Metabolic pathways related to the microbiome metabolism

It has been shown that the gut microbiota has a large effect on the blood metabolome^48,49^, which in turn is independent from the host genetic effect^50^. In this experiment, several metabolites found were related to the gut microbiome metabolism, and their differences indicate changes in the microbial activity of the lines. As mentioned earlier, the secondary bile acids, which included lithocholate, glycolithocolate, and deoxycholic acid glucuronide, were more abundant in the plasma of the L line. Bile acids are polar derivatives of cholesterol and play an essential role in lipid digestion and absorption in the intestine^26^. The secondary bile acids are synthesized from the primary bile acids by the gut bacteria, highlighting the importance of the interactions between the microbiota and the bile acids in energy homeostasis. A metagenomic analysis of the cecum content performed on the 10th generation of selection confirmed the importance of the gut microbiota’s functionality in the IMF deposition; however, no clear association between microbial genes involved in the secondary bile acids formation and the IMF content was found^22^. Further studies are needed to elucidate the role of secondary bile acids in the energy homeostasis of these lines.

The levels of BCAA in plasma discussed above have also been shown to depend on the microbiome composition^51^. In line with the findings of Ridaura et al.^51^, the metagenomic analysis performed on the 10th generation showed an increased abundance of genes involved in the BCAA degradation in the L line, contributing to the decreased circulating BCAA seen in this line and the lower IMF content in the mentioned line^22^. Furthermore, the metabolism of the aromatic amino acids (AAA) tryptophan, tyrosine, and phenylalanine is also related to the microbiome metabolism and was also affected by selection. Greater abundances of the modified amino acid *N*-lactoyl phenylalanine (0.49 SD, *P*_0_ = 0.95), and of metabolites from the tryptophan (0.25–0.78 SD, *P*_0_ ≥ 0.81) and tyrosine (0.56–0.71 SD, *P*_0_ ≥ 0.97) metabolisms were found in the H line. Together with the BCAA, the AAA can be metabolized either by the host or by the gut microbiota^52^, and they have been shown to be increased in obesity^53^. In this experiment, most of the metabolites from the AAA metabolism found were directly or indirectly gut-derived, except the kynurenine, which is a product of the tryptophan catabolism through the host’s kynurenine pathway. This pathway is also used for the biosynthesis of the cofactor NAD, and its precursor, the nicotinamide, was also more abundant in the plasma of the H line (0.57 SD, *P*_0_ = 0.98). Finally, metabolites from the histidine metabolism were also more abundant in the plasma of the H line (0.30–0.74 SD, *P*_0_ ≥ 0.85). Among those metabolites, the imidazole propionate was found, which is produced by gut-microbiota. The imidazole propionate can modulate host inflammation and metabolism, it has been positively related to obesity and diabetes^54,55^, and it has even been proposed as a predictor of α diversity^49^.

These results highlight the importance of the microbiome activity in the metabolism of the host and its direct relationship with the determination of the trait. A metabolomic analysis of the gut content of these lines would give a further insight into the microbiome activity and would allow to confirm the results obtained so far.

### Other metabolic pathways affected by selection

Other interesting changes were detected in vitamins A and E metabolisms. Greater abundances of retinol (vitamin A; 0.76 SD, *P*_0_ = 0.99), α-tocopherol (vitamin E; 0.82 SD, *P*_0_ = 1) and β/γ-tocopherol (vitamin E; 0.84 SD, *P*_0_ = 1) were found in the plasma of the H line, together with metabolites from the vitamin E catabolism (α-CEHC and α-CEHC glucuronide; 0.52 to 0.67 SD, *P*_0_ ≥ 0.96). Dietary supplementation of vitamin A has contradictory effects on intramuscular adipose tissue development, promoting the pre-adipocyte hyperplasia and inhibiting the adipocyte final maturation^56^. Since these lines are fed the same diet, the difference found between them must be due to its metabolism, as it was observed in cattle with different feed efficiency^57^. On the other hand, the vitamin E metabolites have important antioxidant activity, and can also act as signaling and gene regulation molecules in pathways related to lipid metabolism^58^. Despite the great number of studies on vitamins A and E, and since they influence many metabolic reactions, it is not clear how their metabolism is affecting the intramuscular fat deposition of these lines. However, these results indicate an interesting association that could be further investigated.

The glycolytic and energy metabolism was also affected by selection. No differences were found between the lines in the plasma glucose concentration neither in previous^24,25^ nor in this experiment. However, greater abundances of metabolites involved in the amino sugar metabolism (0.68 to 1.30 SD, *P*_0_ ≥ 0.99), the fructose, mannose, and galactose metabolism (0.54–1.77 SD, *P*_0_ ≥ 0.97), and the pentose metabolism (0.53 SD, *P*_0_ = 0.96) were found in the plasma of the L line. These results agree with another study performed, in which a greater abundance of lactose was found in the milk of the L line, suggesting an increased metabolism of carbohydrates in the mentioned line (results not published yet). It is not entirely clear the relationship with the intramuscular fat deposition; nonetheless, these results could suggest differences in glucose utilization between the lines.

## Conclusions

The experimental design and the analysis performed in this study allowed us to obtain the genetically determined metabolomic profile of the divergent lines, which is directly related to their intramuscular fat content. The results obtained showed that there was a large and varied response to selection in the plasma metabolome, agreeing with the large polygenic component found in the GWAS previously performed.

The most affected pathways found were those of lipids and amino acids. There was a general increase of lipids in the plasma of the L line, which suggested an impaired β-oxidation in the mentioned line. These results indicated a limited capacity of the L line to obtain energy from lipids, consequently reducing their uptake and re-esterification in muscle and adipose tissue. The most relevant results among the amino acids metabolism were related to the BCAA, which suggested a lower degradation in the gut of the H line, followed by a greater catabolism by the host. Finally, the changes found in the secondary bile acids, in both the branched-chain and aromatic amino acids, and also in the metabolites from the arginine and histidine metabolisms, supported the relevant role of the microbiome activity in the development of the trait, reinforcing the results of the metagenomic analysis previously performed.

## Methods

### Animals

The Research Ethical Committee approved all experimental procedures according to Council Directives 98/58/EC and 2010/63/EU (reference number 2017/VSC/PEA/00212). The rabbits used for this experiment came from two lines divergently selected for intramuscular fat (IMF) content in *Longissimus thoracis et lumborum* (LTL) muscle. The complete selection procedure is described in Martínez-Álvaro et al.^4^. Briefly, the divergent selection started from a base population of 13 sires and 83 does, originating one line with high-IMF content (H) and another with low-IMF content (L). The lines were contemporarily reared under the same environmental conditions, and they were fed the same diet. The litters were weaned at 28 days, then housed collectively and fed *ad libitum* until slaughter with a standard commercial diet containing 16% of crude protein, 16.5% of crude fiber and 2.4% of fat, 7.6% of ashes, 0.8% of calcium, 0.6% of phosphorous, 0.26% of sodium; and supplemented with vitamin A (10,000 UI/kg), vitamin D3 (900 UI/kg), vitamin E (25 mg/kg), iron (78 mg/kg), cobalt carbonate (0.30 mg/kg), manganese (20 mg/kg), zinc (50 mg/kg), selenium (0.05 mg/kg), potassium iodide (1.0 mg/kg), copper (8 mg/kg), and bacitracin zinc antibiotic (100 ppm). The fatty acid composition of the diet, expressed as a percentage of total fatty acids was: 54.8% of C18:2n6, 19.7% of C18:1n9, 16.6% of C16:0, 5.7% of C18:3n3, 1.8% of C18:0, 0.5% of C16:1, and 0.9% of fatty acids with more than 20 carbon atoms.

To perform the selection, two full sibs (one male and one female) of the first parity of each doe were slaughtered at 9 weeks of age and the LTL muscle was excised, minced, and freeze dried. The IMF content was then quantified using near-infrared spectroscopy (NIRS) applying the equations developed by Zomeño et al. (2011)^59^, and expressed as g IMF/100 g of fresh muscle. The does were ranked according to the average IMF value obtained from their offspring, and the top 20% of does provided all females for the next generation. Each sire was mated with six does, the mates were ranked, and only one male progeny from the highest-rated mate of each sire was selected for the next generation to reduce inbreeding.

The rabbit meat is characterized by its low-fat content. The LTL muscle has the lowest fat content^60^, and in these lines it had a mean of 1.06 g IMF/100 g of fresh muscle. The divergent selection performed was successful, achieving a difference in the 9^th^ generation of 0.45 g IMF/100 g of fresh muscle, equivalent to 3.1 standard deviations of the trait^20^.

### Plasma metabolome

The plasma metabolome was quantified in 24 rabbits from the H line (12 males and 12 females) and 24 from the L line (12 males and 12 females) from the 9th generation of selection (see supplementary Data 7). The animals were slaughtered at 9 weeks of age after 4 h of fasting and blood samples from the jugular vein were collected in tubes containing K2 EDTA as anticoagulant (Deltalab, Rubí, Spain). The samples were immediately centrifuged at 3000 rpm for 10 min at room temperature to separate the plasma, which was finally stored at −80 °C. The plasma samples were sent to Metabolon, Inc. laboratory (Morrisville, North Carolina, USA) for their analysis using UPLC-MS/MS: ultra-performance liquid chromatography (ACQUITY UPLC System, Waters) coupled to tandem mass spectrometry (Q-Exactive Orbitrap high resolution/accurate mass, Thermo Scientific) interfaced with a heated electrospray ionization source (HESI-II). The Orbitrap mass analyzer operated at 35,000 mass resolution.

The samples were prepared using the automated MicroLab STAR® system (Hamilton Company, Reno, Nevada, USA). The proteins were precipitated with methanol under vigorous shaking for 2 min (Glen Mills GenoGrinder 2000) followed by centrifugation. The samples were then briefly placed on a TurboVap® (Zymark) to remove the organic solvent and finally stored overnight under nitrogen. Then, four aliquots of each sample extract were taken and reconstituted in solvents compatible to different methodologies: two separate reverse phase (RP)/UPLC-MS/MS with positive ion mode electrospray ionization (ESI), one RP/UPLC-MS/MS with negative ion mode ESI, and one hydrophilic interaction liquid chromatography (HILIC)/UPLC-MS/MS with negative ion mode ESI. These four methodologies will be called positive early, positive late, negative, and polar.

The first aliquot was gradient eluted from a C18 column (Waters UPLC BEH C18-2.1 × 100 mm, 1.7 µm) using water and methanol, containing 0.05% perfluoropentanoic acid and 0.1% formic acid. It was analyzed under acidic positive conditions and chromatographically optimized for more hydrophilic compounds (positive early). The second aliquot was gradient eluted from the same aforementioned C18 column using methanol, acetonitrile, water, 0.05% perfluoropentanoic acid, and 0.01% formic acid. It was analyzed using acidic positive conditions, but chromatographically optimized for more hydrophobic compounds (positive late). The third aliquot was gradient eluted from a separate C18 column using methanol and water, with 6.5 mM Ammonium Bicarbonate at pH 8, and then analyzed using basic negative conditions (negative). The last aliquot was eluted from a HILIC column (Waters UPLC BEH Amide 2.1 × 150 mm, 1.7 µm) using a gradient consisting of water and acetonitrile with 10 mM Ammonium Formate at pH 10.8. Finally, it was analyzed by negative ionization (polar). The MS analysis alternated between MS and data-dependent MSn scans using dynamic exclusion. The scan range varied between methods but covered 70–1000 m/z.

Several types of controls were analyzed together with the experimental samples: a pooled matrix sample generated by taking a small volume of each experimental sample served as a technical replicate throughout the data set; extracted water samples served as process blanks; and a pool of QC standards carefully chosen not to interfere with the measurement of endogenous compounds were spiked into every analyzed sample, to monitor instrument performance and help with the chromatographic alignment.

Raw data were extracted from the four methodologies, obtaining four datasets: positive early, positive late, negative, and polar. The peaks were identified, QC processed, and the compounds were identified by comparison to library entries of purified standards or recurrent unknown entities. The identifications were based on three criteria: retention index, accurate mass match to the library (+/− 10 ppm), and the MS/MS forward and reverse scores between the experimental data and authentic standards. Peaks were quantified using area-under-the-curve. The information regarding function and metabolic pathways of each metabolite identified came from the Human Metabolome Database and the Kyoto Encyclopedia of Genes and Genomes database.

### Data processing

A total of 997 compounds were detected in the plasma of the two rabbit lines with the four methodologies (252 positive-early, 181 positive-late, 448 negative, and 116 polar), from which 852 were known entities. The data processing was performed separately for each dataset.

The metabolites that were undetected (i.e. intensity = 0) in a large number of samples were removed from the datasets. After some exploratory analyses, only metabolites that had less than 10% of zeros in at least one line were kept, and the remaining zeros were imputed using random forest^61^. The quantification of the metabolites in untargeted metabolomics studies refers to relative changes^62,63^, meaning that, as happens with compositional data, the relevant information is contained in the ratios between the parts^64^. Thus, an additive log-ratio transformation^65^ (*alr*) was applied, which is defined as follows:

1 $${{{{{\rm{ln}}}}}}\left(\frac{{x}_{j}}{{x}_{{ref}}}\right)={{{{{\rm{ln}}}}}}\left({x}_{j}\right)-{{{{{\rm{ln}}}}}}({x}_{{ref}}),\,j=1,\,\ldots ,\,J-1,\,j\, \ne \, {ref}$$

where *x*_*j*_ is the abundance of the *j*-th metabolite, *x*_*ref*_ is the abundance of a reference metabolite and *J* is the total number of metabolites in the dataset. The reference metabolite was chosen following the methodology proposed by Greenacre et al. (2021)^23^. Briefly, this methodology consisted in choosing the metabolite that (1) reproduced the geometry of the full set of log-ratios in a Procrustes analysis (measured by the Procrustes correlation), and (2) had low variance, ensuring that the main variation is due to the numerator. After the data processing, the four datasets, corresponding to the four methodologies described earlier, were joined and analyzed as described below.

### Statistics and reproducibility

The metabolic differences between lines were identified by two separate analyses. The first one was a partial least square-discriminant analysis (PLS-DA), for which the dependent variable was a categorical vector coding the H or L line. The second one was a linear partial least square (PLS), for which the dependent variable was a vector containing the IMF content. Both were performed using the R package mdatools^66^ after standardization of the dataset (i.e. subtract the mean and divide by the standard deviation).

**Fig. 5:**
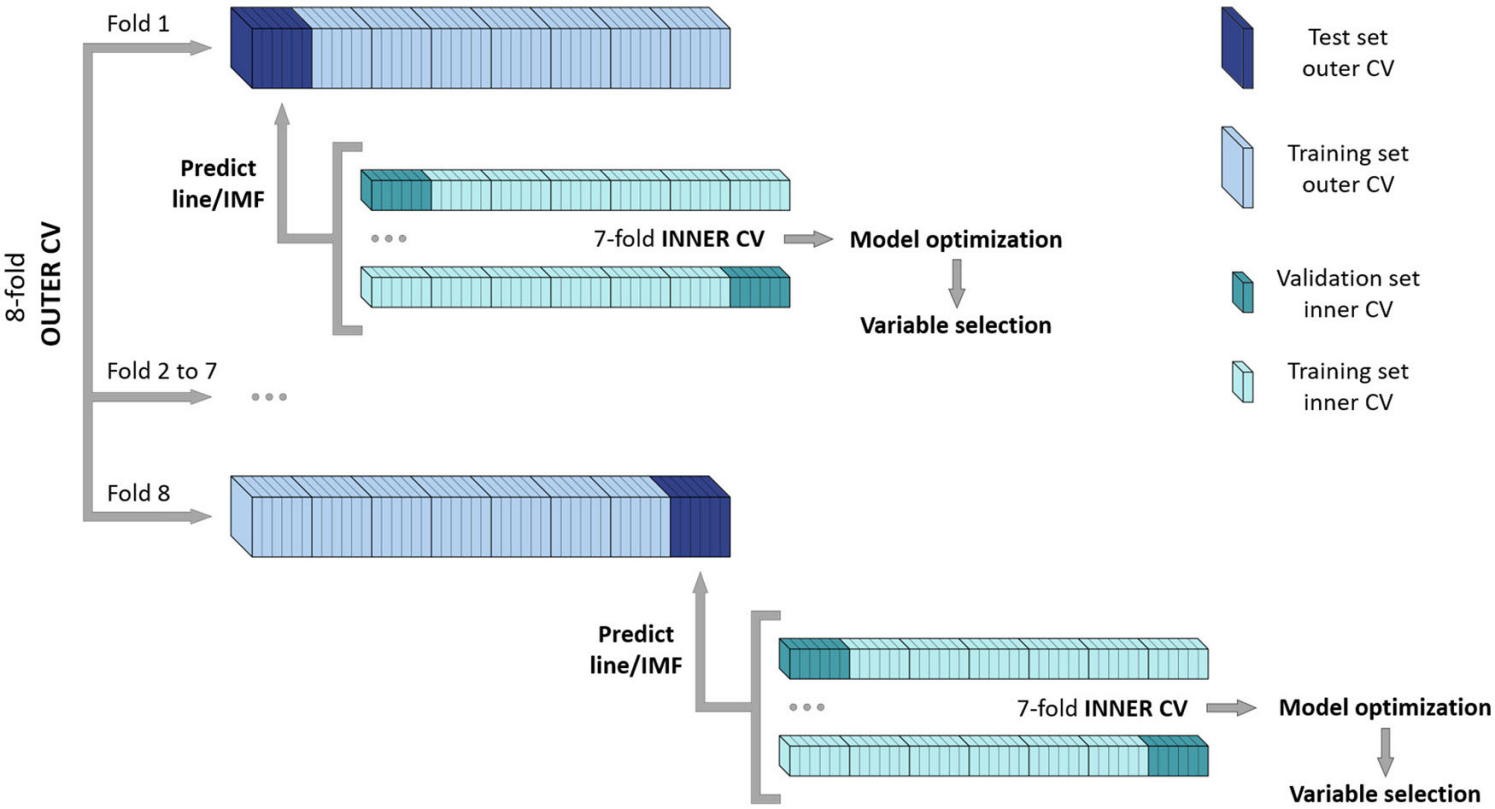
Cross model validation (CMV) diagram. The samples were divided into 8 groups (8-fold outer CV). One group of samples was set aside as a test set and the remaining samples (training set) were subjected to a 7-fold inner CV to develop and optimize the model. Once the model was developed, a variable selection step was performed, and a final model was adjusted. The final model was used to predict the line (in the case of PLS-DA) or IMF content (in the case of PLS) of the group of samples previously set aside. The procedure was repeated 8 times (fold 1–8) until all the groups were in the test set. The whole CMV procedure was repeated 20 times, changing the samples composition of the test and training sets of the outer CV.

A cross model validation (CMV) was performed to avoid overfitting the PLS-DA and PLS models^67^, and to select the relevant metabolites^68^. The CMV consists of an inner cross-validation (CV) performed to develop and optimize the models, and an outer CV to test the models’ performance^67^. After some exploratory analysis, a 7-fold inner CV and an 8-fold outer CV were used. First, the samples were divided into 8 groups of 6 samples each (8-fold outer CV) and one group was set aside as a test set. The remaining samples (training set) were subjected to a 7-fold inner CV procedure to develop and optimize the model. Then, once the model was developed, a variable selection step was performed using the variable importance in projection (VIP) value and the confidence interval of their regression coefficients (CI). Only those metabolites that had a VIP value ≥ 0.8 and which 95% CI did not contain the 0 were kept. Finally, a new model was adjusted with the selected metabolites, and it was used to predict the line (in the case of PLS-DA) or the IMF content (in the case of PLS) of the samples previously set aside as the test set. This procedure was repeated 8 times, until all the groups of the outer CV were set aside once. Additionally, 20 iterations of CMV were performed, changing the composition of the mentioned groups (Fig. 5).

After the CMV, a total of 160 PLS-DA and 160 PLS models were adjusted to predict each test set (20 rounds of 8-fold outer CV). The predictions obtained from the PLS-DA models were used to build an average misclassification table to evaluate the classification ability of the models, while the Q^2^ parameter was used to evaluate the prediction ability of the PLS models. Additionally, a permutation test was performed to analyze if the models’ performance was better than what would be obtained by chance. To perform the test, the values of the dependent variable (H and L classes for the PLS-DA or IMF content for the PLS) were permuted and new models were adjusted, performing the same CMV process mentioned above. The average misclassification table was calculated for the PLS-DA models, while the Q^2^ parameter was obtained for the PLS models^67^.

Once all the models were adjusted and validated, the metabolites considered as relevant were those that were selected in more than 80% of the models (i.e., in more than 128 PLS-DA or PLS models, independently). Finally, only those metabolites selected in both approaches (PLS-DA and PLS) were considered for further analysis.

The sign and magnitude of the differences in metabolite abundances were estimated as the phenotypic difference between lines. The linear model applied was

2 $${{{{{\boldsymbol{y}}}}}}={{{{{\boldsymbol{Xb}}}}}}+{{{{{\boldsymbol{e}}}}}}$$

where ***y*** is the vector of phenotypes, ***b*** is the vector of fixed effects including the line and sex effects, ***e*** is the vector of residual variances, and ***X*** is the incidence matrix. Bayesian inference was applied^69,70^ using the program Rabbit (Institute for Animal Science and Technology, UPV, Valencia, Spain). Bounded flat priors were assumed for all fixed effects and variances. The residual random effects were assumed to be uncorrelated and a priori multinormally distributed as: e ~ N (0, **I**σ^2^_e_).

The marginal posterior distributions of the phenotypic differences between H and L lines were obtained by Gibbs sampling. The parameters of the posterior distributions taken into consideration were the median of the difference (D_H-L_), the highest posterior density interval at 95% (HPD_95%_), and the probability of the difference being greater than zero when D_H-L_ > 0, or lower than zero when D_H-L_ < 0 (*P*_0_)^70^. Additionally, the differences between lines of each metabolite were expressed as units of their standard deviation (SD).

### Reporting summary

Further information on research design is available in the Nature Portfolio Reporting Summary linked to this article.

### Supplementary information

Description of Additional Supplementary Files

Supplementary Data 1–7

Reporting summary

## Data Availability

Source data underlying Fig. 1 and Table 1 are provided in Supplementary Data 1–4. Source data underlying Figs. 2b, 3, and 4 are provided in Supplementary Data 6. Other datasets analyzed during the current study are available from the corresponding author on reasonable request.
