# Supplementary material for: Plasma metabolomic profiling in two rabbit lines divergently selected for intramuscular fat content
Source: Commun Biol. 2023 Aug 31;6:893. doi: 10.1038/s42003-023-05266-3 (PMC10471702; doi:10.1038/s42003-023-05266-3)
Supplement: Supplementary file 1 — Description of Additional Supplementary Files [file 42003_2023_5266_MOESM1_ESM.docx]

**Description of Additional Supplementary Files**

**File name:** Supplementary Data 1

**Description:** Adjustment parameters of the PLS-DA models adjusted using a cross-model validation procedure.

**File name:** Supplementary Data 2

**Description:** Adjustment parameters of the PLS-DA models adjusted with permuted data using a cross-model validation procedure.

**File name:** Supplementary Data 3

**Description:** Adjustment parameters of the PLS models adjusted using a cross-model validation procedure.

**File name:** Supplementary Data 4

**Description:** Adjustment parameters of the PLS models adjusted with permuted data using a cross-model validation procedure.

**File name:** Supplementary Data 5

**Description:** Complete plasma metabolomic profile obtained from the IMF divergent lines.

**File name:** Supplementary Data 6

**Description:** Results from the statistical analysis of the 322 relevant metabolites selected in both approaches (PLS-DA and PLS models)

**File name:** Supplementary Data 7

**Description:** Information of the samples used in the untargeted metabolomic analysis
